# Development and application of metallo-phthalocyanines as potent G-quadruplex DNA binders and photosensitizers

**DOI:** 10.1007/s00775-023-02003-3

**Published:** 2023-07-14

**Authors:** Ariadna Gil-Martínez, Adrián Hernández, Cristina Galiana-Roselló, Sònia López-Molina, Javier Ortiz, Ángela Sastre-Santos, Enrique García-España, Jorge González-García

**Affiliations:** 1 Institute of Molecular Science (ICMol) and Department of Inorganic Chemistry, University of Valencia, C./Jose Beltran 2, 46980 Paterna, Spain; 2 Área de Química Orgánica, Instituto de Bioingeniería, Universidad Miguel Hernández, Avda. de la Universidad s/n, 03202 Elche, Spain

**Keywords:** G-Quadruplex DNA, Nickel phthalocyanine, Photosensitizer, Zinc phthalocyanine, Metallo-phthalocyanines

## Abstract

Metallo-phthalocyanines (**MPc**) are common photosensitizers with ideal photophysical and photochemical properties. Also, these molecules have shown to interact with non-canonical nucleic acid structures, such as G-quadruplexes, and modulate oncogenic expression in cancer cells. Herein, we report the synthesis and characterisation of two metallo-phthalocyanines containing either zinc (**ZnPc**) or nickel (**NiPc**) in the central aromatic core and four alkyl ammonium lateral chains. The interaction of both molecules with G-quadruplex DNA was assessed by UV–Vis, fluorescence and FRET melting experiments. Both molecules bind strongly to G-quadruplexes and stabilise these structures, being **NiPc** the most notable G-quadruplex stabiliser. In addition, the photosensitizing ability of both metal complexes was explored by the evaluation of the singlet oxygen generation and their photoactivation in cells. Only **ZnPc** showed a high singlet oxygen generation either by direct observation or by indirect evaluation using a DPBF dye. The cellular evaluation showed mainly cytoplasmic localization of **ZnPc** and a decrease of the IC_50_ values of the cell viability of **ZnPc** upon light activation of two orders of magnitude.

**Graphical abstract:**

**Figure:**
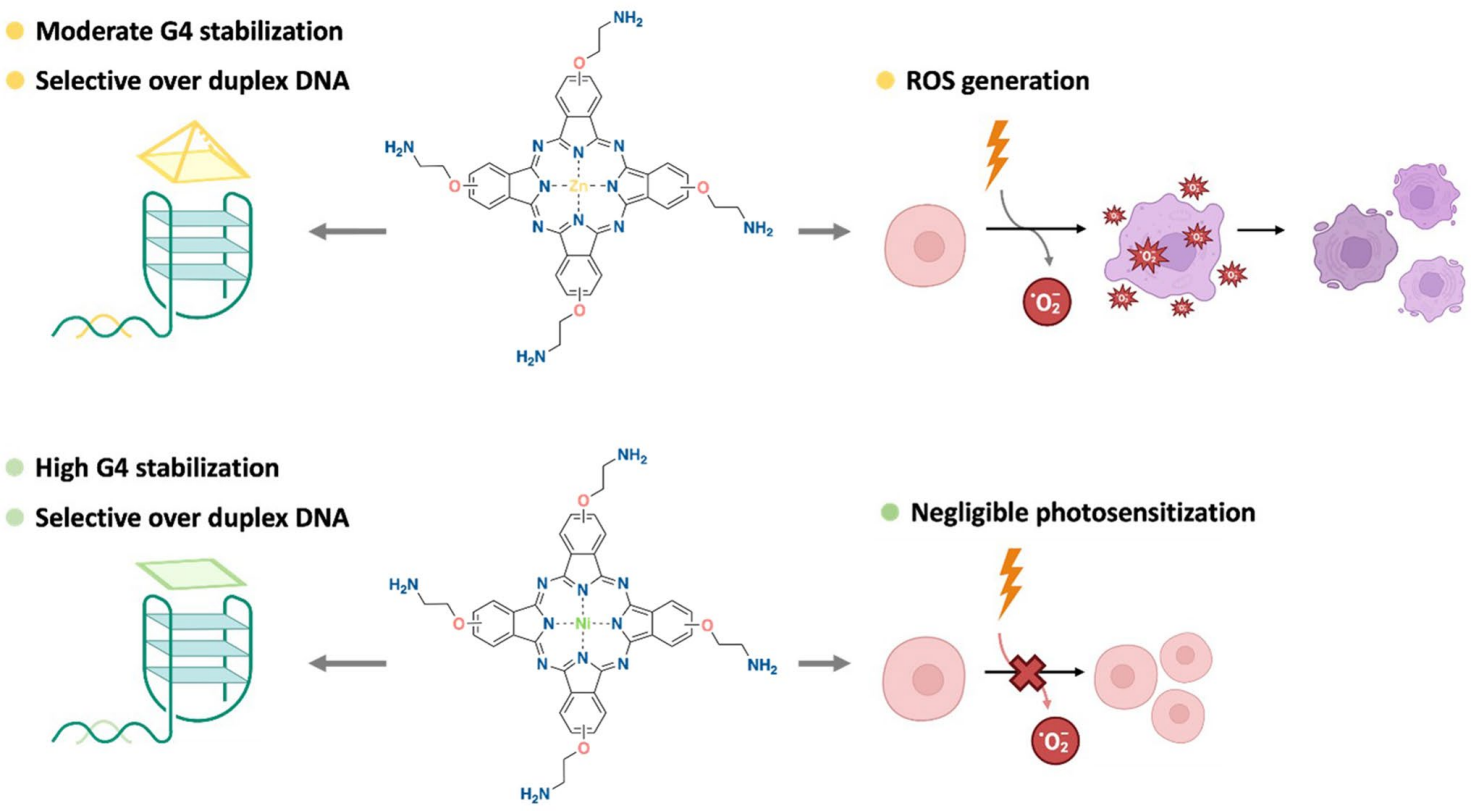
Two metallo-phthalocyanines containing zinc and nickel within the aromatic core have been investigated as G-quadruplex stabilizers and photosensitizers.** NiPc** shows a high G4 binding but negligible photosensitizing ability while** ZnPc** exhibits a moderate binding to G-quadruplex together with a high potency to generate singlet oxygen and photocytotoxicity. The interaction with G4s and capacity to be photosensitized is associated with the geometry adopted by the central metal core of the phthalocyanine scaffold.

**Supplementary Information:**

The online version contains supplementary material available at 10.1007/s00775-023-02003-3.

## Introduction

Modern medicinal chemistry covers the discovery and targeting of novel disease modulators, such as histone modifications, nucleosome remodellers, modified DNA/RNA bases and a variety of non-coding mediating elements [1]. One of the most attractive non-coding targets in cancer and neurodegenerative disorders is G-quadruplex (G4s) nucleic acid structure. [2–4] G4s are non-canonical DNA or RNA structures formed by guanine-rich sequences, which form planar rearrangements of four guanine bases termed as G-quartets [5, 6]. These guanine quads result from the hydrogen bonding association between the Hoogsteen and Watson–Crick faces of the guanines and the metal coordination of potassium and sodium to the guanine oxygens. [5, 6] Next-generation sequencing and bioinformatic analysis have located putative G4s forming sequences in telomeres, oncogene promoter regions, replication initiation sites and untranslated regions in human genomes [7]. Of outmost importance is the evidence of G4s formation under physiological conditions in cells and its key role in regulating biological processes, such as oncogene expression, telomere maintenance and chromosome stability, [8] which highlight the potential of using G4s as anticancer targets by small molecules [9–19]. In this regard, G4 binders Quarfloxin and CX-5461 have reached clinical trial stages for the treatment of cancers, and other drugs, allegedly targeting G4s, are currently under the preclinical scope [20, 21]. Quarfloxin inhibits the RNA polymerase through the interaction with ribosomal G4s in the nucleolus, resulting in a reduction of the tumour volume in pancreatic cancer xenograft models, but advanced clinical studies have withdrawn it due to bioavailability issues [22]. CX-5461 has a complex mechanism involving the stabilisation of the promoters of *cMyc, cKit* proto-oncogenes and telomere G4s in addition to the blockage of the replication forks, which results in the induction of DNA damage and inhibition of ribosomal RNA biogenesis [23]. Currently, it is evaluated in phase I clinical trials for patients with BRCA1/2 deficient tumours [24].

Recently, an interesting approach to tackle cancer has emerged combining G4 binding and the photosensitization of the G4 ligand, which can then generate reactive oxygen species (ROS) and the resulting breakage of the G4 DNA/RNA structures and other nearby biomolecules. In this line, a family of porphyrins was photosensitized to cleave G4 RNAs from the Rat sarcoma virus (*ras*) oncogenes, which then reduced the tumour growth in pancreatic xenograft models [25]. In most of the works, porphyrins have been selected as G4 ligands with photosensitizing properties[26–29], but the large efforts in the last years to apply photodynamic therapy into the clinics have generated novel molecules which overcome the limitations of traditional photosensitizers (poor aqueous solubility, poor photostability…) [30–35]. The main attention has focussed on the incorporation of a metal ion into an aromatic core. In this regard, metallo-phthalocyanines are a promising family scarcely explored as dual G4 binders / photosensitizers. MPcs harbour the large π-planar structure able to interact via π–π stacking with the external tetrads of G4s and the appropriate photophysical properties to act as photosensitizer in photodynamic therapy (i.e. Q band in the visible range for irradiation, high quantum yield and lifetime, etc.). [36, 37] Their capacities for G4 binding and photosensitizing strongly depend on the coordinated metal. Zinc(II) phthalocyanines promote significant photosensitivity although adopt a non-planar metal coordination that hampers G4 interaction in contrast to Ni(II) square planar metallo-phthalocyanines which have more idoneous structures for G-tetrad stacking but promote low photosensitivity [38, 39]. Several important G4 ligands are described by Luedtke et al*.* based on guanidinium and amide-modified zinc phthalocyanines. [40–42]. Amongst them, the zinc phthalocyanine Zn-DIGP, substituted with isopropylguanidines showed excellent affinity and stabilisation effect towards G4 DNAs from *cMyc*, *cKit, KRAS* and *HTelo* with high selectivity for G4s over double- and single-stranded DNA. Strikingly, Zn-DIGP exhibited both turn-on luminescence upon G4 binding and down-regulation of *c-myc* and *KRAS* expression in cancer cell lines, suggesting a G4-mediated promoter inhibition [40, 41]. Several nickel and zinc metallo-phthalocyanines containing eight quaternary ammonium groups have shown large HTelo G4 stabilisation effect and potent inhibition of the telomerase [43, 44]. Other phthalocyanines containing copper or gallium have demonstrated the G4 binding [45, 46] with a concomitant suppression of the photo-generation of ROS [47]. Miyoshi group evaluated the G4 binding and the photo-irradiation consequences of several metallo-phthalocyanines, suggesting that the down-regulation of *ras* expression is due to the G4 binding in the oncogene promoter with the concomitant selective photocleavage [48].

**Fig. 1:**
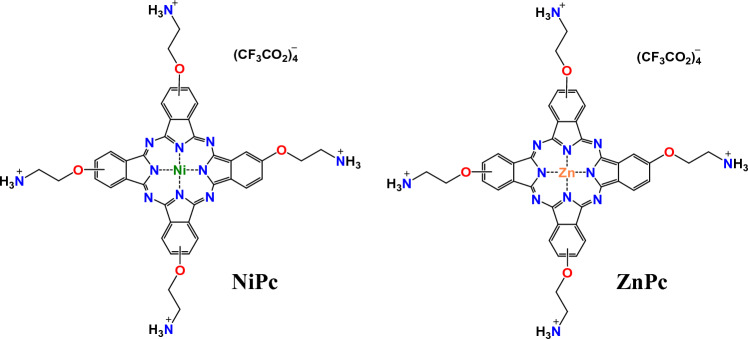
Metallo-phthalocyanines studied in this work

Herein, we report the preparation of two metallo-phthalocyanines incorporating zinc (**ZnPc**) and nickel (**NiPc**) within the aromatic core and containing four ethylammonium trifluoroacetate (TFA) substituents in the non-peripheral positions (Fig. 1). The photo-physical properties and the aggregation behaviour of these molecules have been studied by UV–Vis/fluorescence emission experiments. Their interaction with G4s and duplex DNAs has been assessed using FRET melting assays and UV–Vis and fluorescence emission spectroscopies. Then, the singlet oxygen generation has been studied in vitro by means of different biophysical methods. Lastly, the viability in several cell lines has been evaluated in the dark and using red light.

## Experimental methods

All solvents and reagents were purchased from commercial sources and used as received. ^1^H /^13^C NMR spectra were recorded with a Bruker Avance 400 spectrometer, using tetra-methyl-silane as a reference. The solvent for spectroscopic studies was of spectroscopic grade and used as received. UV–Vis spectra were measured with Helios Gamma and Cary 100 UV–Vis spectrophotometers. Fluorescence spectra were recorded with HORIBA scientific SAS and PTI spectrophotometers. High-resolution mass spectra were obtained from a Bruker Microflex LRF20 matrix-assisted laser desorption/ionisation time-of-flight (MALDI-TOF). IR spectra were measured with Nicolet Impact 400D spectrophotometer. The unlabelled and labelled DNA oligonucleotides (see Table S1) were purchased from IDT DNA purified in HPLC grade, and the labelling dyes were 5’-FAM (FAM: 6-carboxyfluorescein) and 3’-TAMRA (TAMRA: 6-carboxytetramethylrhodamine). All concentrations of oligonucleotides were estimated by UV absorption using the extinction coefficients and expressed in strand molarity. Ligands were dissolved in DMSO to give 5 mM stock solutions. All solutions were stored at − 20 °C and defrosted and diluted immediately before use in the suitable buffer to the appropriate concentrations.

### Synthesis of NiPc-Boc

**Scheme 1:**
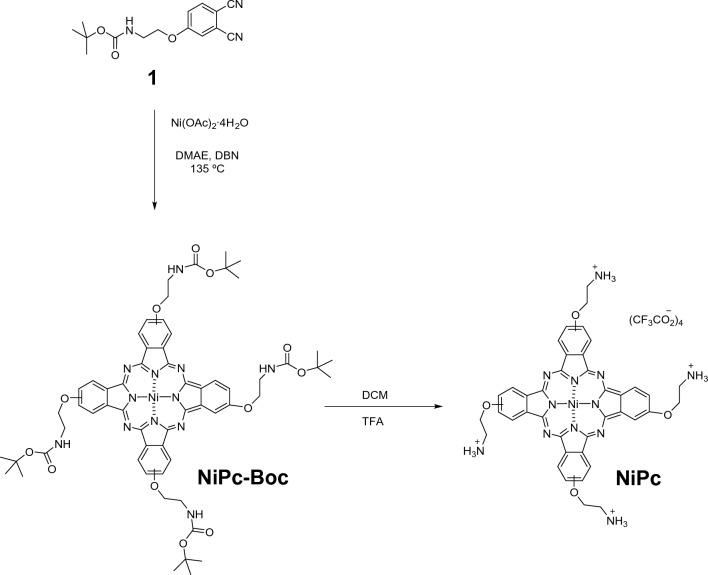
Synthetic route of **NiPc**

300 mg (1.04 mmol) of 4-(2-*tert*-butoxycarbonylaminoethoxy)phthalonitrile (**1**, see Scheme 1), 129.91 mg (0.52 mmol) of Ni(OAc)_2_·4H_2_O, and DBN, 1,5-Diazabicyclo[4.3.0]non-5-ene (DBN, 3 drops) in di-methyl-amino-ethanol (DMAE, 600 µL) were stirred at 135 ºC for 7 h under an inert atmosphere. The blue mixture was cooled at room temperature, concentrated under vacuum and purified by column chromatography (DCM:MeOH 94:6) yielding 50 mg (24%) of **NiPc-Boc**. HR-MALDI-TOF (dithranol): *m/z* for C_60_H_68_N_12_NiO_12_: calcd. 1206.4433 [M^+^]; found, 1206.4589. UV–Vis (DMF) λ_max_/nm (log ε): 382 (4.37), 613 (4.53), 674 (4.92). IR (ν_max_/cm^−1^): 3370, 2976, 2933, 2877, 1704, 1612, 1531, 1462, 1419, 1393, 1366, 1351, 1272, 1242, 1171, 1129, 1096, 1070, 963, 894, 868, 823, 782, 752, 651, 582.

### Synthesis of NiPc

50 mg (0.04 mmol) of **NiPc-Boc** was dissolved in a mixture of DCM (1 ml) and TFA (1 ml) and stirred for 2 h at 0ºC. The blue mixture was heated at room temperature and concentrated under vacuum, yielding **NiPc** quantitatively. HR-MALDI-TOF (dithranol): *m/z* for C_40_H_36_N_12_NiO_4_: calcd. 806.2336 [M^+^]; found, 806.2322. UV–Vis (H_2_O) *λ*_max_/nm (log ε): 370 (4.03), 625 (4.40). IR (ν_max_/cm^−1^): 3294, 2929, 2879, 1729, 1655, 1608, 1531, 1456, 1394, 1336, 1280, 122, 1123, 1091, 1062, 1014, 960, 837, 749, 700, 622. ^1^H NMR (TFA-*d*_1_): *δ* = 4.13 (br s, 8H, CH_2_O), 5.04 (br s, 8H, CH_2_N), 8.01 (br s, 4H, ArH), 9.11 (br s, 4H, ArH) 9.48 (br s, 4H, ArH).

### FRET melting assay

Labelled DNA was dissolved as a 20 µM stock solution in MilliQ water and then a solution of 400 nM concentration was prepared in cacodylate buffer (pH 7.3) supplemented with potassium or sodium. The solutions were annealed at 95 °C for 10 min, and allowed to cool slowly to room temperature overnight. The buffer used for the antiparallel G4 HTelo was 100 mM NaCl, 10 mM LiCac, whilst for the rest of G4s and duplex was 100 mM KCl, 10 mM LiCac. Ligand solutions were diluted from stock solutions (see above) to a final concentration of 20 μM in the buffer. Each well of a 96-well plate (Applied Biosystem) was prepared with 60 µl, with a final 200 nM DNA concentration and increasing concentration of tested ligands (0–4 µM). Measurements were performed on a PCR AriaMx (Agilent Technologies) with excitation at 450–495 nm and detection at 515–545 nm. Readings were taken from 25 to 95 °C at interval of 0.5 °C maintaining a constant temperature for 30 s before each reading. Each measurement was done in triplicate. The normalised fluorescence signal was plotted against the compound concentration and the Δ*T*_m_ values were determined.

### Spectrophotometric and spectrofluorimetric titrations

The DNA oligonucleotides were dissolved in Tris buffer (100 mM KCl, 10 mM Tris pH 7.4) and annealed at 95 °C for 10 min before cooling to room temperature overnight. The concentration of DNA was confirmed using the molar extinction coefficients provided by the manufacturer. Annealing concentrations were approximately 500 µM. Absorption spectra were recorded with a Cary 100 UV–Vis Spectrometer (Agilent) in quartz cells (path length 1 cm) using scan rates of 300 nm min.^−1^. Fluorescence spectra were recorded with a PTI Spectrofluorimeter in quartz cells with a cross-section of 1 × 0.5 cm, using slit widths of 2 nm and an integration time of 0.1 s. The fluorescence emission spectra were recorded between 630 and 900 nm with an excitation wavelength of 620 nm. UV–Vis titrations were conducted with a concentration of **MPc** of 5 µM whilst fluorescence titrations used 2 µM. The absorption/emission maxima data were analysed according to the independent-site model by means of a Levenberg–Marquardt fitting routine and equations reported previously by Thordarson. [49]

### Singlet oxygen evaluation

*Indirect evaluation:* Singlet oxygen generation was evaluated in air using the indirect method with di-phenyl-isobenzofuran (DPBF) acting as a singlet oxygen chemical quencher in DMSO. To avoid chain reactions of the quencher in the presence of singlet oxygen, the concentration of DPBF was kept at ∼3 × 10^−5^ M. Solutions of the **MPc** with an absorbance of ∼0.5 at the irradiation wavelength were prepared in the dark and irradiated at 730 nm with a LED array lamp in the presence of DPBF. Then, the reactions were followed spectrophotometrically by observing the decrease in the 417 nm absorption peak of DPBF as a function of irradiation time. *Direct evaluation:* The sample was prepared in an air-saturated acetonitrile or deuterated water solution with an absorption of 0.2 at 400 nm. The sample was irradiated at 400 nm with a mounted M450LP1 LED (Thorlabs). To cut off light at wavelengths shorter than 850 nm, a long-pass glass filter was placed in front of the monochromator entrance slit. The signal was detected with an EO-817L IR-sensitive liquid-nitrogen cooled germanium diode detector (North Coast Scientific Corp.). The luminescence signal, centred at 1270 nm, was measured from 1100 to 1400 nm.

### Cell culture

HeLa Human cervical and A549 lung cancer cells and RAW 264.7 macrophages were grown in low glucose Dulbecco’s modified Eagle medium containing 10% foetal bovine serum at 37 °C with 10% CO_2_ in humidified air. Cells were kept continuously under confluence before split twice a week and the possibility of contamination was excluded by performing regularly mycoplasma tests.

### Cellular imaging

Cells were seeded on chambered cover glass (*ca.* 2 × 10^4^, 300 μl, 0.8 cm^2^) for 24 h, then the medium was replaced with fresh phenol red-free medium containing **ZnPc** (20 μM, 200 μl) for 2 h. Prior to imaging, the cells were washed with PBS and replaced with fresh growth medium. Cells were imaged using a confocal fluorescence microscope (FV1000, Olympus). Using a 60 × magnification microscope objective (water immersion, NA ¼ 1.2) and an excitation wavelength of 620 nm for **MPc**, images of the cells were recorded in both transmission and fluorescence modes. For the fluorescence images, the detection band was 640–800 nm which covered the emission range of the metal complexes.

### Intracellular oxidative stress

The measurement of the broad spectrum of intracellular ROS gave an indicative assessment of the oxidative stress using the specific oxidation-sensitive fluorescent probe 2′,7′-dichlorofluorescein diacetate (DCFH-DA). Cells were loaded with DCFH-DA (25 µM) diluted in serum-free medium and incubated at 37 °C for 60 min. Then, they were treated with 10, 50 and 100 µM solutions of **MPc** for 24 h. The fluorescence of the probe was registered in a multi-mode microplate reader with an excitation wavelength of 485 nm and an emission wavelength of 528 nm before and after irradiation at 730 nm during 20 min (5.2 mW cm^−2^, 3.1 J cm^−2^) light using an Atlas Photonics LUMOS BIO irradiator.

### Phototoxicity on cell cultures

A total of 5 × 10^3^ HeLa, A549 and RAW 264.7 cells were seeded on 96-well plates and allowed to adhere for 24 h. The cells were treated with increasing concentrations of the **MPc** diluted in cell medium achieving a total volume of 200 μl. The cells were incubated with the **MPc** for 24 h and, then the medium was refreshed with phenol red-free medium. To study the phototoxic effect of the **MPc**, the cells were exposed to 730 nm (spectral half-width: 20 nm, 10 min, 5.2 m W cm^−2^, 3.1 J cm^−2^) light using an Atlas Photonics LUMOS BIO irradiator. To study the dark cytotoxicity of the **MPc**, the cells were not irradiated and the medium exchanged. The cells were grown for an additional 24 h period at 37 °C. After this time, the medium was replaced with fresh medium containing MTT with a final concentration of 0.5 mg mL^−1^. The cells were incubated for 4 h and the generated formazan crystals were solubilised in 100 μl DMSO. The absorbance was registered with a SpectraMax M2 Microplate Reader (Molecular Devices). The obtained data was analysed with the GraphPad Prism software.

## Results and discussion

### Design, synthesis and photophysical characterisation of the metallo-phthalocyanines

Attending to the two main structural characteristics of potent G4 binders, comprising a planar polycyclic π-deficient core and one or several charged side chains, we prepared two metallo-phthalocyanines differentiated by the metal within the macrocyclic isoindole core, either nickel or zinc. In the nickel phthalocyanine, the metal coordinates four nitrogen atoms of the macrocycle adopting a square planar geometry whilst zinc generates a pyramidal geometry by coordinating an additional solvent molecule in addition to the nitrogen atoms of the macrocycle. The different arrangement will allegedly impact the binding to G4s through the most common binding mode of *π–π* stacking on the top of the G-quartets. The metal complexes were also designed with four side chains with pH-dependent protonable groups to enhance the aqueous solubility and hamper the self-aggregation. [43, 50, 51] Moreover, these moieties can improve the interaction with DNA by binding to the phosphates and the nucleobases.

**ZnPc** was synthesised according to the literature, with a minor modification on the procedure to obtain the TFA salt instead of the HCl one [52]. **NiPc-Boc** was obtained upon the cyclo-tetramerization of the precursor phthalonitrile **1** in the presence of Ni(OAc)_2_ (see Scheme 1) and further purification by chromatography (*yield:* 24%). The ^1^H NMR spectra registered in different deuterated solvents (data not shown) showed no well-defined signals due to aggregation of the phthalocyanine core. Then, **NiPc** was obtained in quantitative yield by treatment of **NiPc-Boc** with TFA (see Scheme 1). **NiPc** was characterised by ^1^H NMR and FT-IR spectroscopies and HR-MALDI-TOF spectrometry. The ^1^H NMR spectrum in TFA-*d*_*1*_ showed well-defined aromatic and aliphatic signals: three signals from the isoindole units can be found at 9.48, 9.11 and 8.01 ppm integrating for 12 hydrogen atoms, and from the aminoethoxy chain two signals can be observed, one at 5.04 corresponding to the 8 protons closer to the amine group and the second one at 4.13 ppm corresponding to the 8 protons closer to the oxygen (Figure S1). Additionally, HR-MALDI-TOF assays, performed at positive mode confirmed the obtention of both, **NiPc-Boc** and **NiPc**, with isotopic distributions that match the simulated isotope patterns. Furthermore, in the FT-IR spectrum of **NiPc,** the band at 1704 cm^−1^ corresponding to the carbonyl group (C = O) of the **NiPc-Boc** is missing, as well as the apparition of a broad band of the ammonium groups centred at 3294 cm^−1^ (Figures S3 and S4).

Both **MPc** show the typical UV–Vis bands of metallo-phthalocyanines with a band at lower wavelength and centred at 350 nm, which is assigned to the Soret band of the phthalocyanine scaffold (see Table 1). A second band located within the visible region and centred at 680 nm is ascribed to the Q band. The linearity of the absorption versus the concentration of the metal complexes indicates the absence of aggregation in aqueous solution up to 100 µM for **ZnPc** and 50 µM for **NiPc** (Figure S5). Similar to other metallo-phthalocyanines, only the zinc phthalocyanine presents a fluorescence emission band at 705 nm in H_2_O (see Table 1).

**Table 1:** Photophysical data of the two metallo-phthalocyanines (**MPc**) in different solvents

MPc	Solvent	*λ*_abs_ (nm)	Ɛ (cm^−1^ M^−1^)	*λ*_em_ (nm)
NiPc	H_2_O	625	25,370	
NiPc	CH_3_CN	600	21,978	
ZnPc	H_2_O	637	8083	705
ZnPc	CH_3_CN	627	6186	

### Interaction of the metallo-phthalocyanines with DNAs

**Fig. 2:**
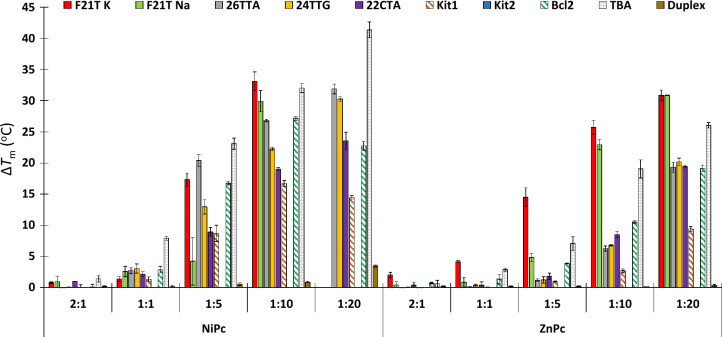
Representation of Δ*T*_m_ values obtained from the FRET melting studies for the interaction of the metallo-phthalocyanines with several G4/duplex DNA structures. The concentration of DNA was 0.2 μM, whereas the concentration of the **MPc** was increased and the [DNA]:[**MPc**] ratios showed in the bottom part (2:1, 1:1, 1:5, 1:10 and 1:20). Errors denote the standard deviations of at least three independent experiments

We initially evaluated the stabilisation of DNA induced by the ligands by FRET melting experiments. We included G4s of different topology (parallel, antiparallel and mixed/hybrid) and number of G-tetrads (e.g. 2 and 3 tetrads), as well as a duplex model (see Table S1). **NiPc** induced a larger stabilisation effect for all G4s than **ZnPc** (i.e. Δ*T*_m_ values in F21T-K G4 are 33.1 and 25.7 for **NiPc** and **ZnPc**, respectively at ratio [DNA]:[**MPc**] of 1:10). The larger stabilisation produced by **NiPc** can be ascribed to the square planar geometry of the nickel core, which can stack more efficiently on the top of the external G-tetrads in comparison with the pyramidal geometry of the zinc site with less efficient stacking. Both ligands do not stabilise the duplex model (dark brown bar in Fig. 2), indicating a high degree of selectivity for G4 over duplex DNA. The low interaction of the **MPc** towards double-stranded DNA structures has already been observed and associated to the large aromatic core, which hampers the base pair intercalation and the groove binding to the double helix [40, 41, 50, 51, 53]. Strikingly, **NiPc** induces a high stabilisation effect on the *bi*tetrad thrombin G4 TBA (light grey bar in Fig. 2).

**Fig. 3:**
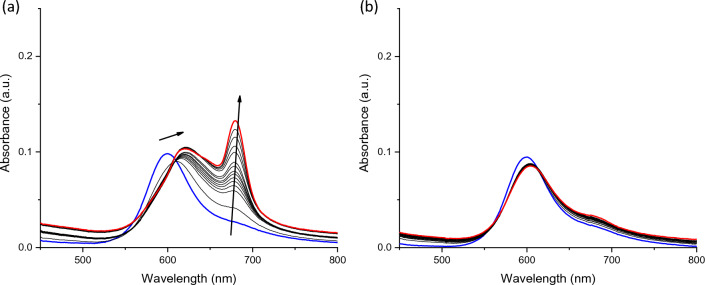
UV–Vis titrations of **NiPc** with cMyc G4 **a**, and ds26 **b**. Blue spectrum corresponds to the initial titration and red spectrum to last one

Once assessed the stabilisation effect, we performed UV–Vis titrations with HTelo G4 (telomeric region), cMyc G4 (oncogene promoter) and a duplex DNA (ds26) to evaluate the affinity for DNAs. **NiPc** and **ZnPc** present in aqueous solution an absorption band at 600 and 625 nm, respectively, assigned to the *Q* band (Fig. 3). Addition of DNA to **ZnPc** yields a decrease in the band and the appearance of a new Vis band at 690 nm (Figures S6–S11). The intensity of this band depends on the structure studied, being G4s the structures that show the largest enhancement of the intensity. **NiPc** shows a decrease and a red shift of the visible band in addition to the apparition of a new band centred at 720 nm when bound to G4s (Fig. 3). UV–Vis titrations with duplex DNA afforded minimal change in these bands, indicating a soft interaction for double-stranded DNA in concordance with the FRET melting results. The affinity constants calculated from the titrations are collected in Table 2, which indicate the high affinity for G4s of both **MPc** (*K*_a_ ≈ 10^6^ M^−1^). In contrast, both complexes show low affinity for the duplex DNA (*K*_*a*_ < 10^3^ M^−1^), confirming the selectivity observed by the FRET melting assays.

**Table 2:** Binding constants (log K_a_) determined from the UV–Vis and fluorescence titration experiments of MPc with G4 and duplex DNAs in Tris buffer (100 mM KCl, 10 mM Tris pH 7.4).

	HTelo	cMyc	ds26
NiPc	5.2 (± 0.1)^1^	6.3 (± 0.2)^1^	n.d
ZnPc	6.7 (± 0.4)^1^ 5.2(± 0.1)^2^	6.3 (± 0.7)^1^ 6.3 (± 0.2)^2^	n.d 4.6 (± 0.4)^2^

*nd* no determined

^1^Obtained by UV–Vis titrations. ^2^Obtained by fluorescence titrations

**Fig. 4:**
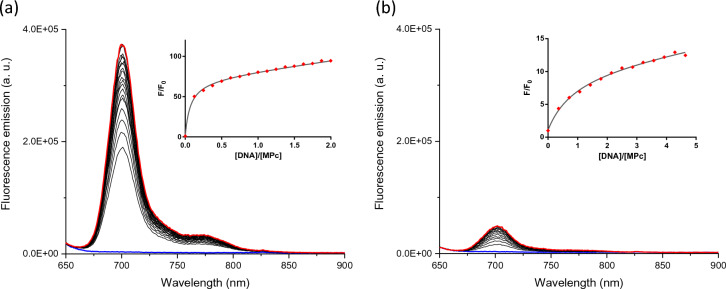
Fluorescence titrations of **ZnPc** with G4 HTelo **a** and ds26 **b** in Tris 10 mM, KCl 100 mM, pH 7.4, λ_exc_ = 620 nm. Inset: Plot of the F/F_o_ *vs.* ratio [DNA]/[**MPc**]. Blue spectrum corresponds to the initial titration and red spectrum to last one

Fluorescence emission titrations were carried out to determine the binding affinity of **ZnPc** for different DNAs. **ZnPc** shows a low fluorescent emission in aqueous-buffered conditions, and the addition of DNAs yields the apparition of a fluorescent emission band centred at 702 nm (see Fig. 4 and S12–S16 in ESI). The intensity changes of the fluorescence emission are different depending on the nucleic acid structure, being larger for G4s (G4-DNA yields a 60-fold increase whilst duplex DNA experiences a 20-fold increase, see Fig. 4). The binding constants afforded values within the micromolar range for G4s structures (see Table 2) whilst the duplex model presents affinity constants of two orders of magnitude lower, which agrees with the UV–Vis and FRET melting experiments.

### Assessment of the ROS generation

**Fig. 5:**
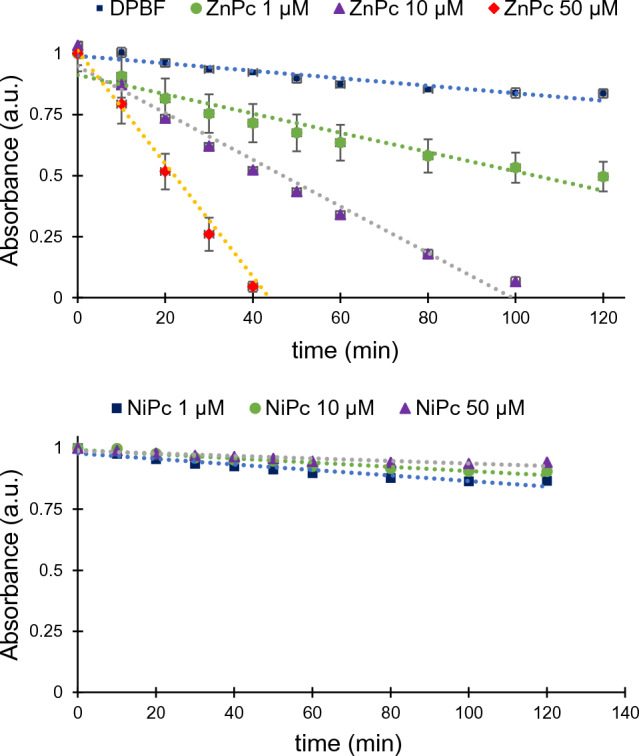
Comparison of the decay rate of DPBF in DMSO induced by **ZnPc** (top panel) and **NiPc** (bottom panel) under red light irradiation

Following our initial hypothesis, the capacity to regulate gene expression of G4 ligands can be tightly associated to the photosensitizing capacity which breaks down the G4 structures upon binding. Therefore, we assessed the capability to induce singlet oxygen under irradiation of both metallo-phthalocyanines and well-known photosensitizers, Zinc 2,9,16,23-tetra-*tert*-butyl-29*H*,31*H*-phthalocyanine (**Zn-PS**) and Rose Bengal (**RB**). We studied the DPBF quenching upon irradiation of the **MPc** at different concentrations and irradiation times. DPBF traps selectively singlet oxygen (^1^O_2_) and arises a decrease in the band at 460 nm, whose intensity is directly associated to the ^1^O_2_ generation. As it can be observed in Fig. 5, **ZnPc** generates singlet oxygen even at the lowest concentration studied (1 µM), being the generation of ^1^O_2_ dependent on the concentration of the metallo-phthalocyanine. In contrast, the **NiPc** barely generates ^1^O_2_ species indicating that the metal core is fundamental for the activation of oxygen radicals upon irradiation. Interestingly, the quenching rates of DPBF are higher for **ZnPc** than for **Zn-PS** (Figure S17) indicating that **ZnPc** is a more efficient photosensitizer than the reference photosensitizer **Zn-PS**.

An additional assessment was conducted by measuring the phosphorescent spectra of the singlet oxygen generated by the **MPc** and **RB** upon irradiation. We could only observe phosphorescence for **ZnPc** and **RB** in acetonitrile solution (Figure S18), suggesting that they can act as photosensitizers in contrast to **NiPc** which is unable to generate ^1^O_2_. The intensity of the spectrum of **ZnPc** is higher than that of **RB**, indicating a qualitative estimation of the higher oxygen sensitising effect of **ZnPc**.

### Cellular experiments using the metallo-phthalocyanines

**Fig. 6:**
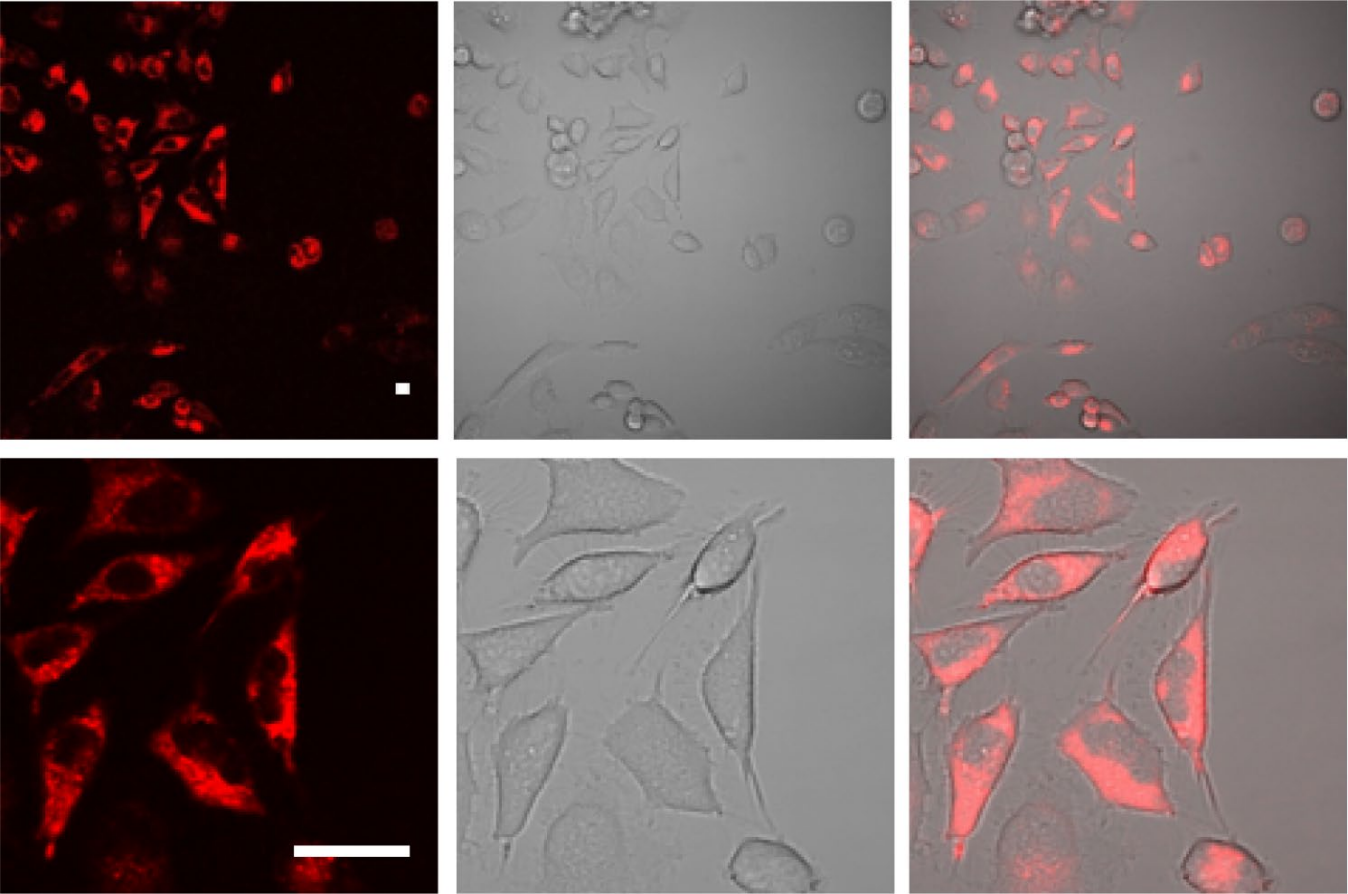
Confocal fluorescence images of HeLa cells incubated with **ZnPc** (20 µM, 2 h). Left panel is the fluorescence emission of **ZnPc**, central panel is bright field and right panel is the image emerged from the fluorescence and bright field. Scale bar: 20 µm

Initially, we investigated the cell localization of **ZnPc** by confocal fluorescence microscopy using its intrinsic fluorescence emission. The metal complex was rapidly taken up into human cervical cancer cells (HeLa) as a bright red emission was detected in the cytoplasm of the cells (Fig. 6).

**Fig. 7:**
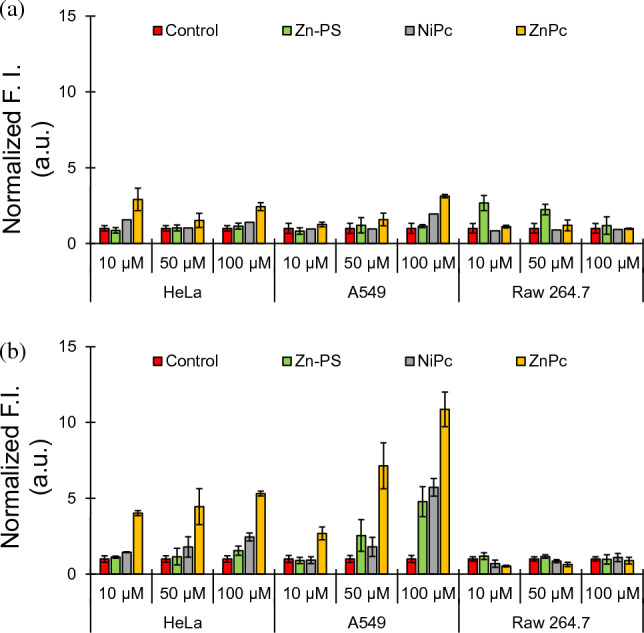
ROS levels in HeLa, A549 and Raw 264.7 cells treated with **NiPc**, **ZnPc** and **Zn-PS** at different concentrations (10, 50 and 100 µM). Top panel in the dark and bottom panel irradiated at 730 nm

Once confirmed that **ZnPc** enters into the cells, we assessed the oxidative stress generated on HeLa, lung cancer cells (A549) and macrophages (Raw 264.7) by both **MPc** in the dark and upon the irradiation at 730 nm. The quantification of the ROS was evaluated by monitoring the cleavage of the intracellular fluorescence probe DCFH-DA (see Experimental Methods). We included the broadly used **Zn-PS** as a positive control of photosensitizer and H_2_O_2_. The incubation of the **MPc** with cells in the dark has not significant increase of the ROS except for the **ZnPc** at the highest concentration (100 µM, 24 h), with an enhancement to 3.5- and 2.2-fold of the initial cellular ROS (Fig. 7, top).

In contrast, cells treated with both **MPc** and irradiated at 730 nm during 20 min generated a significant amount of ROS. Cells treated with **NiPc** increased up to 2.4- and 5.7-fold ROS depending on the cell line. The zinc phthalocyanine treatment yielded over 2.5-fold increase of ROS for the lowest concentration, reaching to 100-fold ROS increase using the largest concentration. **Zn-PS** showed a similar trend than **ZnPc** although with lower quantity of ROS generated, suggesting that **ZnPc** has higher capacity to generate ROS than reference metallo-phthalocyanine photosensitizer.

**Fig. 8:**
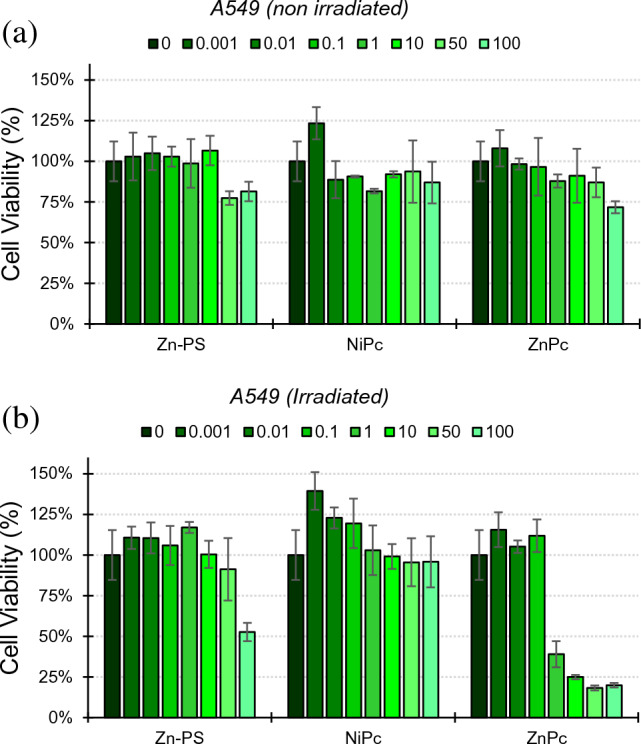
Cell viability on A549 cell line of **Zn-PS**, **NiPc** and **ZnPc** in the dark **a** and under irradiation **b**. Colours and numbering in the legend refer to the compound concentrations (µM)

Then, the photocytotoxic potential of the **MPc** was evaluated on the same cell lines. **Zn-PS** was also included as a control in our experiments. The results are summarised in Table 3 and representative dose–response plots are shown in Figs. 8 and S19–S22. The cells were incubated with increasing concentrations of compounds for 24 h, irradiated at 730 nm for 20 min or maintained in the dark and then, incubated for an additional 24 h, before determining the cytotoxicity. As shown in Table 3, very low toxicity (IC_50_ values > 50 μM) was obtained for **NiPc** as well as for **ZnPc** in the dark. In contrast, **ZnPc** shows high toxicity when irradiated with IC_50_ values ranging from 0.04 µM to 0.89 µM depending on the cell line. These results confirm the capacity of **ZnPc** to exert photocytotoxicity under red light irradiation.

**Table 3:** Values of IC_50_ assessed by MTT of the **MPc** in the dark and under irradiation

Cell line	NiPc		ZnPc		Zn-PS	
	Dark	Irrad	Dark	Irrad	Dark	Irrad
HeLa	> 100	> 100	> 100	0.039	> 100	> 100
A549	> 100	> 100	> 100	0.89	> 100	> 100
Raw264.7	> 100	> 100	58.7	0.37	22.9	42.4

## Conclusions

Two metallo-phthalocyanines have been obtained in good yield, which incorporate zinc and nickel within the aromatic core and containing four trifluoroacetate ethylammonium substituents in the non-peripheral positions, termed as **ZnPc** and **NiPc** respectively. The photophysical properties and the aggregation behaviour of the molecules show no aggregation of both molecules in water, indicating that the positively charged ammonium groups at physiological pH increase the aqueous solubility and helping to disrupt the molecule inter-aggregation. Both metallo-phthalocyanines show affinity constants in the micromolar range for G4s by means of UV–Vis and fluorescence emission experiments, whereas the interaction to duplex DNA is lower, *ca.* *K*_a_ = 10^–3^ M. In line with photophysical studies, FRET melting assays show a large stabilisation effect on G4s of **NiPc** and moderate stabilisation effect of **ZnPc**, suggesting that the central metal core is important for G4 binding and that the square planar geometry of nickel has a better π–π overlapping with the G-quartets than that of the pyramidal geometry of zinc complex. In addition, no one stabilises the duplex DNA, in agreement with UV–Vis and fluorescence titrations.

As photosensitizers, **ZnPc** exhibits a higher singlet oxygen generation upon red light activation in vitro and in cellular assays in contrast to **NiPc** which shows negligible photosensitization. Strikingly, the ROS generation of **ZnPc** is comparable to photosensitizers, such as **RB** and **Zn-PS**. Finally, the treatment of the HeLa, A549 and Raw 264.7 cells with **MPc** shows a strong photocytotoxic effect for **ZnPc**, having IC_50_ values five orders of magnitude lower than in the dark. The results herein described give new insights into the development of novel anticancer drugs operating via a dual mechanism involving G4 binding and photokilling of the cancer cells.

## Supplementary Information

Below is the link to the electronic supplementary material.

Supplementary file1 (PDF 1147 KB)

## Data Availability

Data will be made available on request.

## Abbreviations

Pc: Phthalocyanine
MPc: Metallo-phthalocyanine
G4: G-quadruplex
Quad: Quadruplet
DBN: 1,5-Diazabicyclo[4.3.0]non-5-ene
DMAE: Dimethylaminoethanol
DCFH-DA: 2′,7′-Dichlorofluorescein diacetate
ROS: Reactive Oxygen Species
Zn-PS: Zinc 2,9,16,23-tetra-tert-butyl-29H,31H-phthalocyanine
RB: Rose Bengal
