# Supplementary material for: Development and application of metallo-phthalocyanines as potent G-quadruplex DNA binders and photosensitizers
Source: J Biol Inorg Chem. 2023 Jul 14;28(5):495–507. doi: 10.1007/s00775-023-02003-3 (PMC10368564; doi:10.1007/s00775-023-02003-3)
Supplement: Supplementary file 1 — Supplementary file1 (PDF 1147 KB) [file 775_2023_2003_MOESM1_ESM.pdf]

Development and Application of Metallo-Phthalocyanines as Potent G-quadruplex DNA Binders and Photosensitizers.

Ariadna Gil-Martínez,[a] Adrián Hernández,[b] Cristina Galiana-Roselló,[a] Sònia López-Molina,[a] Javier Ortiz,[b] Angela Sastre-Santos[b,] Enrique García-España,[a] Jorge González-García[a]*

[a]Institute of Molecular Science (ICMol) and Department of Inorganic Chemistry, University of Valencia, C./ Jose Beltran 2, 46680 Paterna, Spain

[b]Área de Química Orgánica, Instituto de Bioingeniería, Universidad Miguel Hernández, Avda. de la Universidad s/n, 03202 Elche, Spain

Table S1. List of DNA sequences used in this work, acronym, localization in the genome

Abreviation	Sequence (5' → 3')	Topology	Localization
**HTelo**	AGGGTTAGGGTTAGGGTTAGGG	Mixed	telomere
**F21T-K**	GGGTTAGGGTTAGGGTTAGGG	Mixed	telomere
**F21T-Na**	GGGTTAGGGTTAGGGTTAGGG	Antiparallel	telomere
**CEB25**	AAGGGTGGGTGTAAGTGTGGGTGGGT	Parallel	minisatellite loci
**Kras**	AGGGCGGTGTGGGAATAGGGAA	Parallel	promoter of *ras*
**22CTA**	AGGGCTAGGGCTAGGGCTAGGG	Antiparallel	telomere
**26TTA**	TTAGGGTTAGGGTTAGGGTTAGGGTT	Hybrid 2	telomere
**24TTG**	TTGGGTTAGGGTTAGGGTTAGGGA	Hybrid 1	telomere
**Kit1**	AGGGAGGGCGCTGGGAGGAGGG	Parallel	promoter of kit
**Kit2**	CGGGCGGGCGCGAGGGAGGGG	Parallel	promoter of kit
**Bcl2**	GGGCGCGGGAGGAATTGGGCGGG	Hybrid	promoter of Bcl2
**TBA**	GGTTGGTGTGGTTGG	Antiparallel	Thrombin binding aptamer
**ds26**	CAATCGGATCGAATTCGATCCGATTG	B-type duplex	

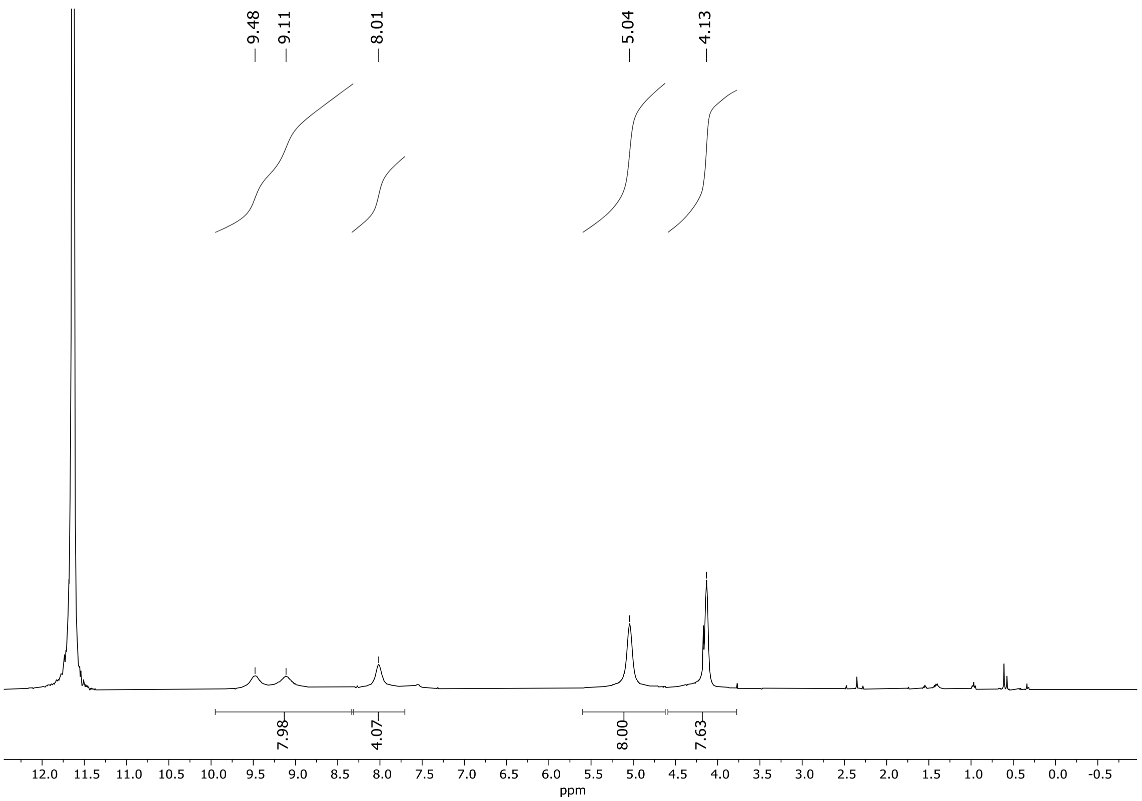

Figure S1.- ^{1}H NMR spectrum of **NiPc** in TFA-*d*

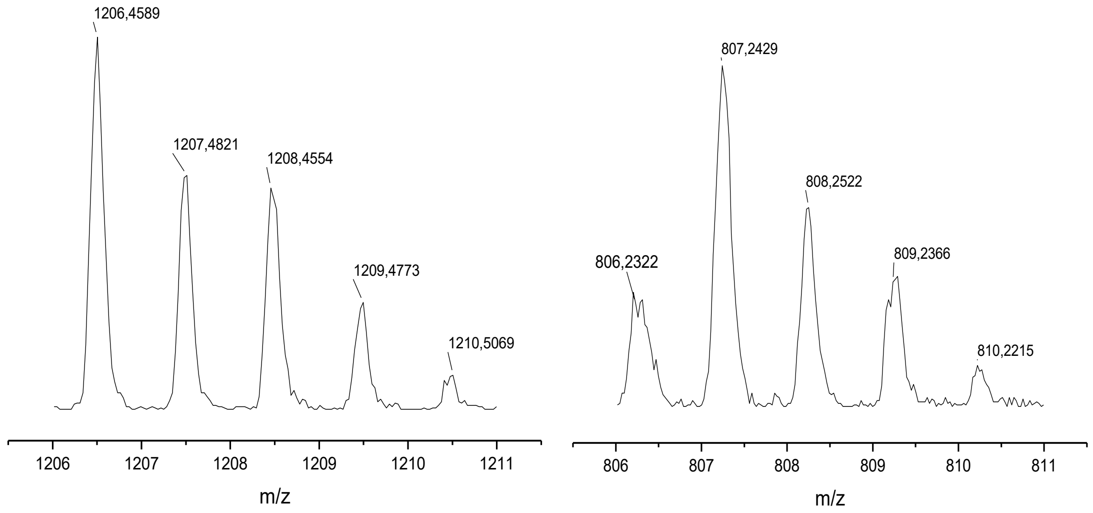

Figure S2. HR-MALDI-TOF spectrum of **NiPc-Boc** (left panel) and **NiPc** (right panel).

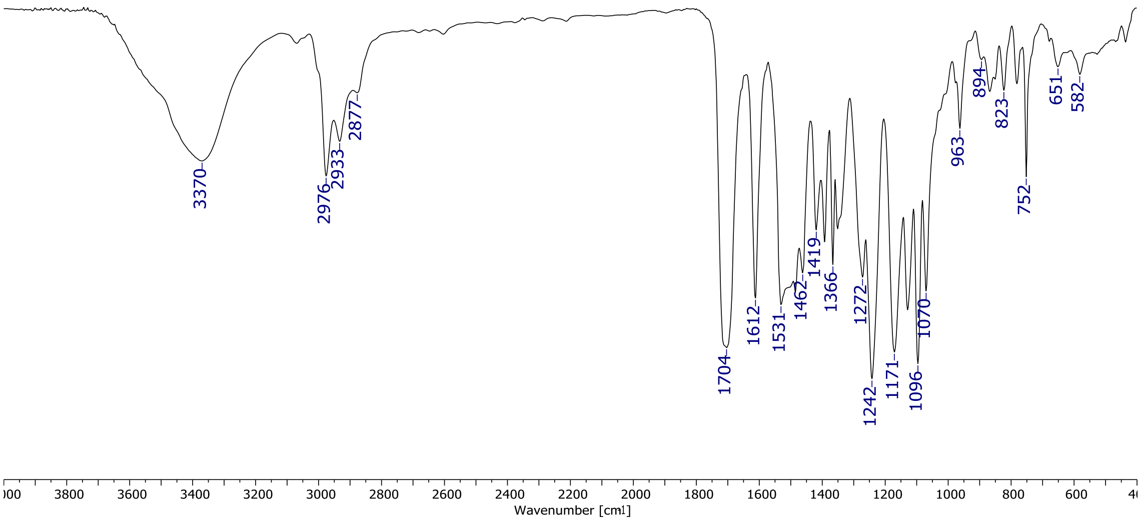

Figure S3. FT-IR spectrum of **NiPc-Boc**

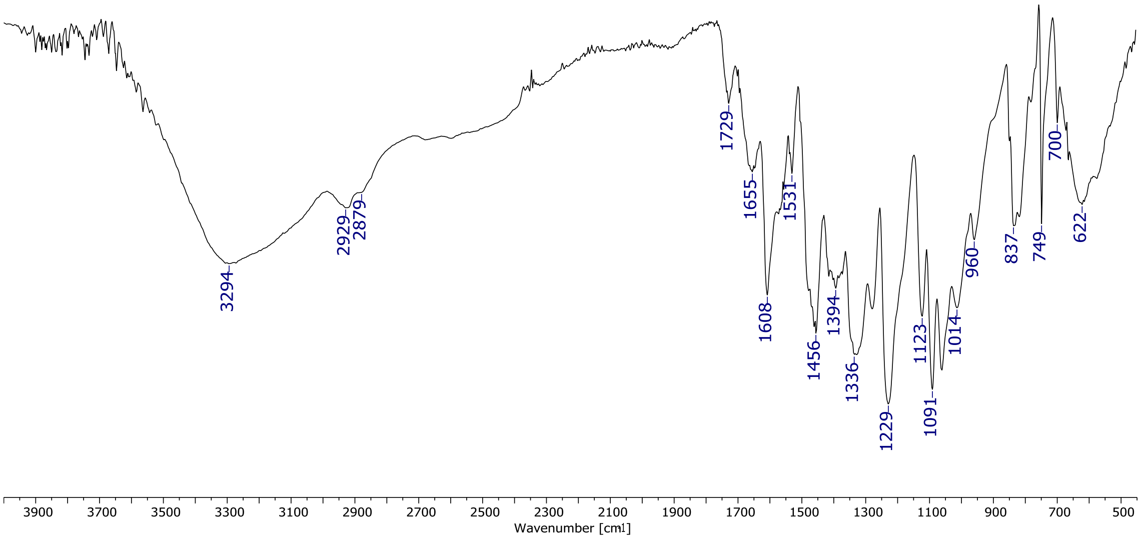

Figure S4. FT-IR spectrum of **NiPc**

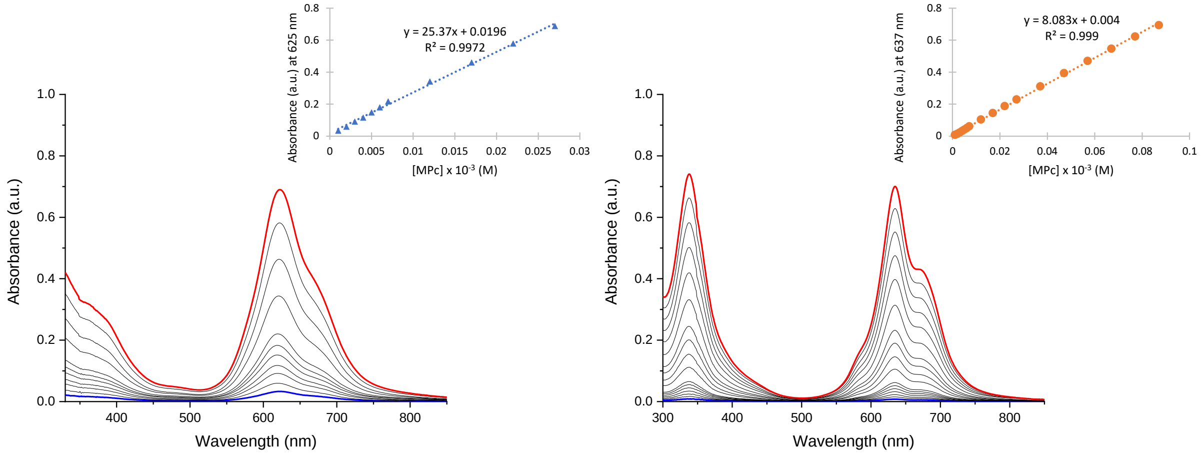

Figure S5.- UV-Vis spectra of **NiPc** (left panel) and **ZnPc** (right panel) at different concentration (1 μM – 100 μM) in H_2O. Insets: Lambert-Beer Plots for MPcs. Blue spectrum corresponds to the initial titration and red spectrum to last one.

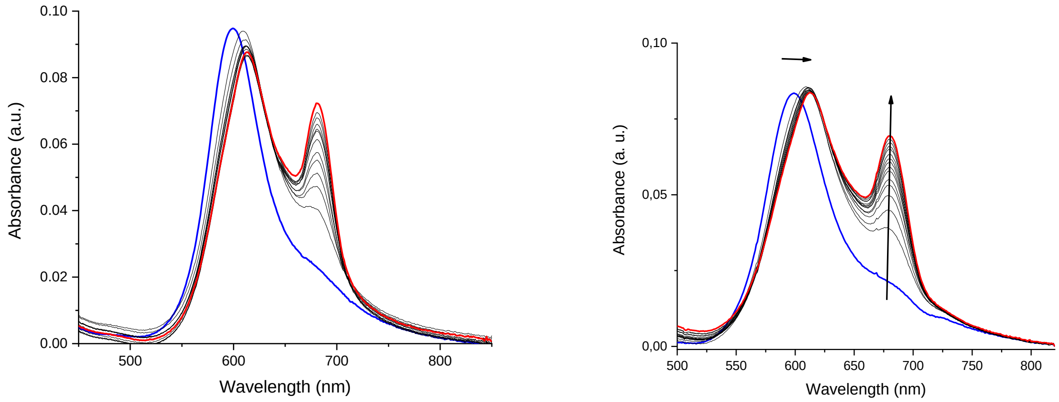

Figure S6. UV-vis titration of **NiPc** (5 μM) with G4 DNA HTelo22 in Tris buffer (Tris 10 mM, KCl 100 mM) at pH= 5 (left panel) and pH 7.4 (right panel). Blue spectrum corresponds to the initial titration and red spectrum to last one.

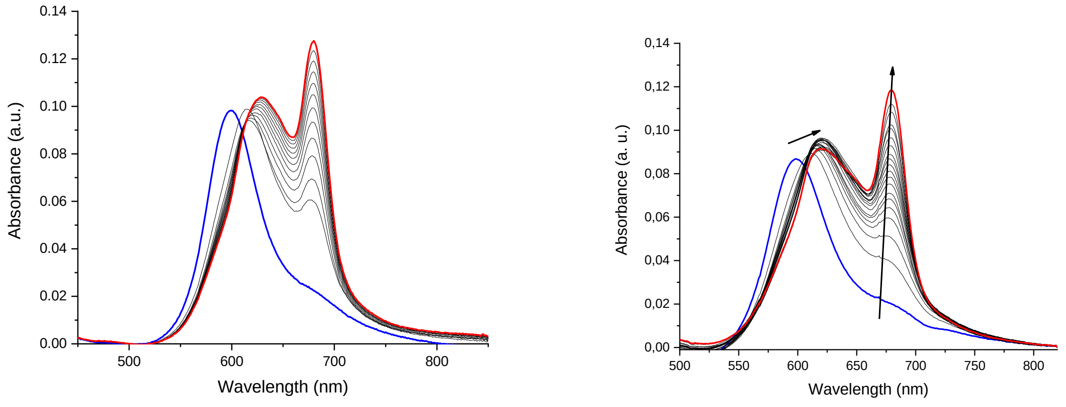

Figure S7. UV-vis titration of **NiPc** (5 μM) with G4 DNA cMyc in Tris buffer (Tris 10 mM, KCl 100 mM) at pH= 5 (left panel) and pH 7.4 (right panel). Blue spectrum corresponds to the initial titration and red spectrum to last one.

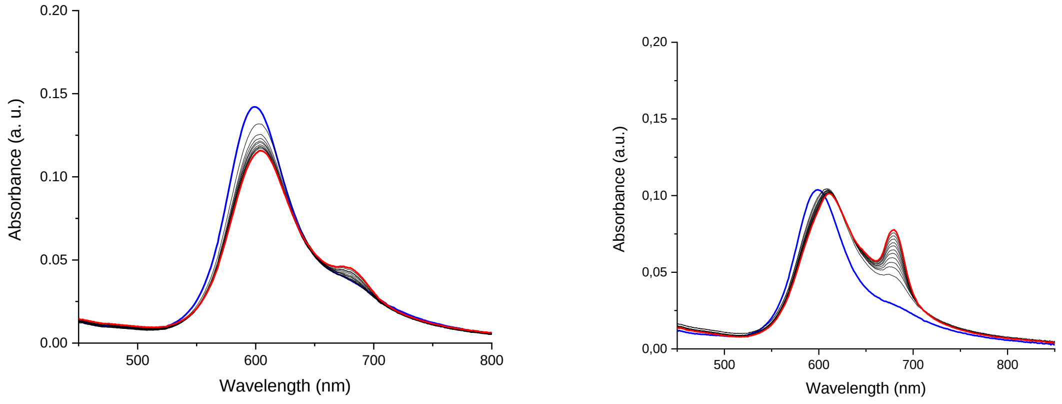

Figure S8. UV-vis titration of **NiPc** (5 μM) with duplex DNA ds26 in Tris buffer (Tris 10 mM, KCl 100 mM) at pH= 5 (left panel) and pH 7.4 (right panel). Blue spectrum corresponds to the initial titration and red spectrum to last one.

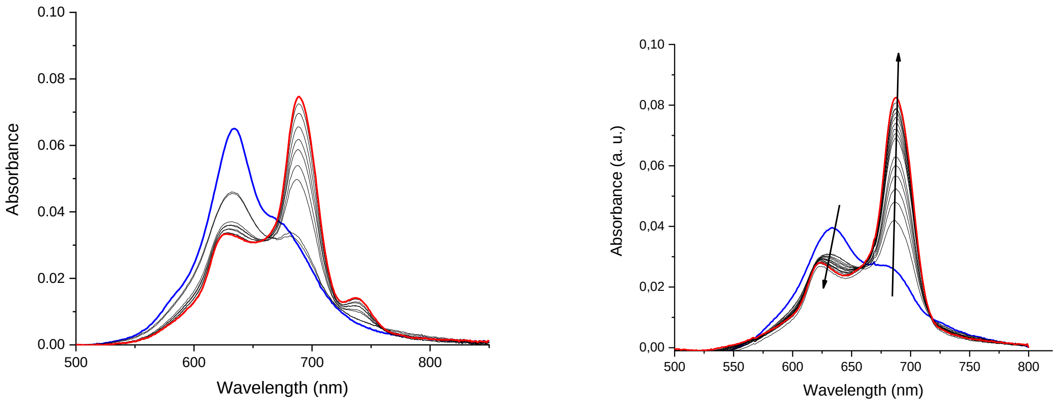

Figure S9. UV-vis titration of **ZnPc** (5 μM) with G4 DNA HTelo22 in Tris buffer (Tris 10 mM, KCl 100 mM) at pH= 5 (left panel) and pH 7.4 (right panel). Blue spectrum corresponds to the initial titration and red spectrum to last one.

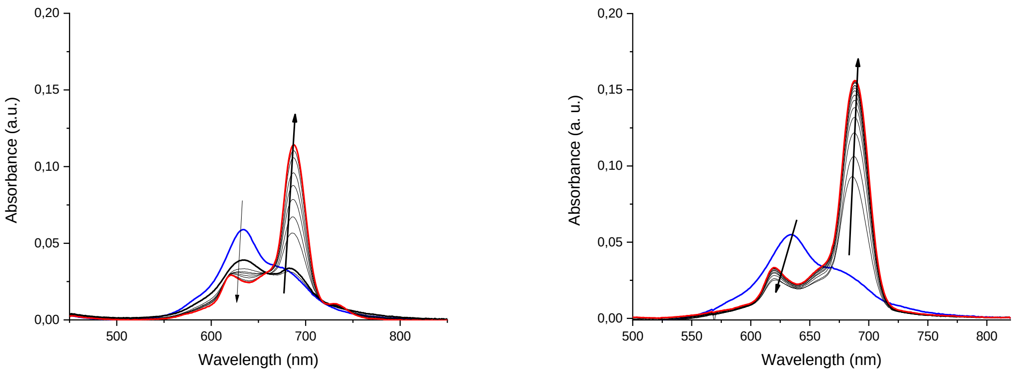

Figure S10. UV-vis titration of **ZnPc** (5 μM) with G4 DNA cMyc in Tris buffer (Tris 10 mM, KCl 100 mM) at pH= 5 (left panel) and pH 7.4 (right panel). Blue spectrum corresponds to the initial titration and red spectrum to last one.

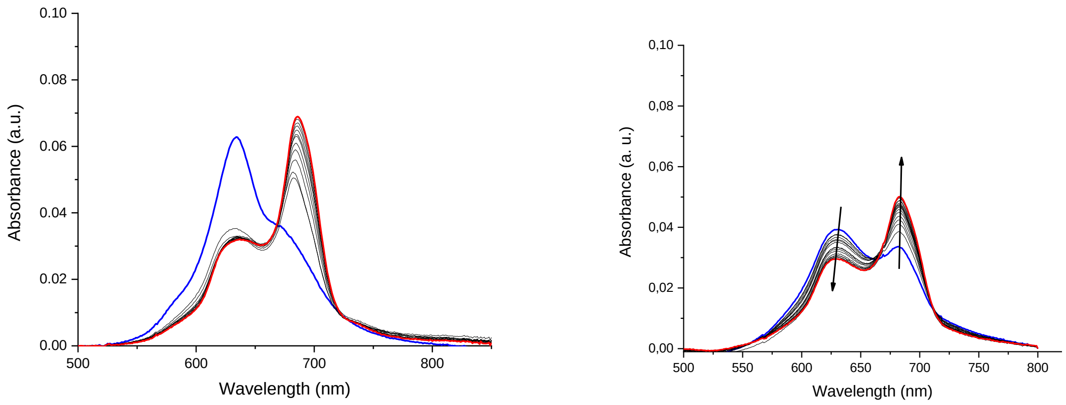

Figure S11. UV-vis titration of **ZnPc** (5 μM) with duplex DNA ds26 in Tris buffer (Tris 10 mM, KCl 100 mM) at pH= 5 (left panel) and pH 7.4 (right panel). Blue spectrum corresponds to the initial titration and red spectrum to last one.

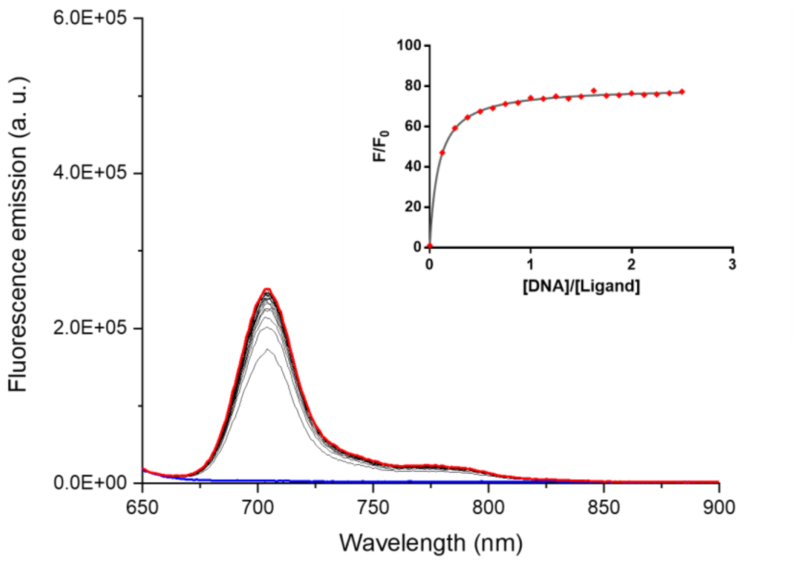

Figure S12. Fluorescence titration of **ZnPc** (2 μM) with G4 DNA HTelo in Tris buffer (Tris 10 mM, KCl 100 mM, pH= 7.4, λ_{exc} = 620 nm). Inset: Plot of the F/Fo vs. ratio [DNA]/[MPc]. Blue spectrum corresponds to the initial titration and red spectrum to last one.

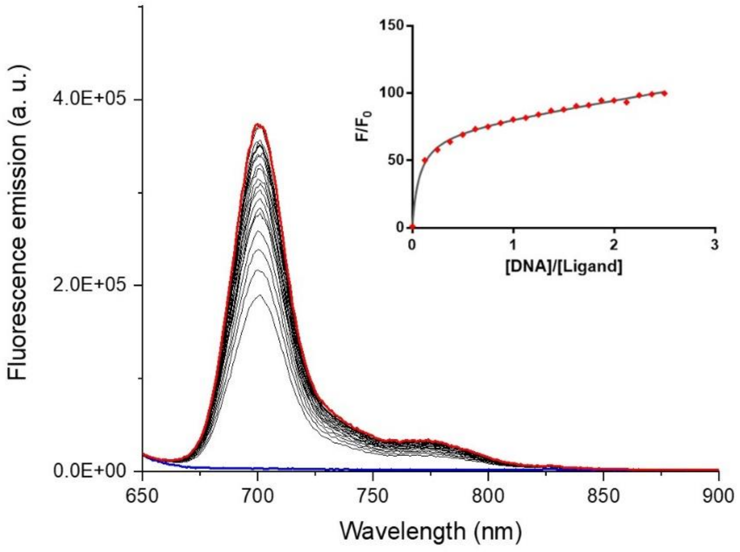

Figure S13. Fluorescence titration of **ZnPc** (2 μM) with G4 DNA cMyc in Tris buffer (Tris 10 mM, KCl 100 mM, pH= 7.4, λ_{exc} = 620 nm). Inset: Plot of the F/Fo vs. ratio [DNA]/[MPc]. Blue spectrum corresponds to the initial titration and red spectrum to last one.

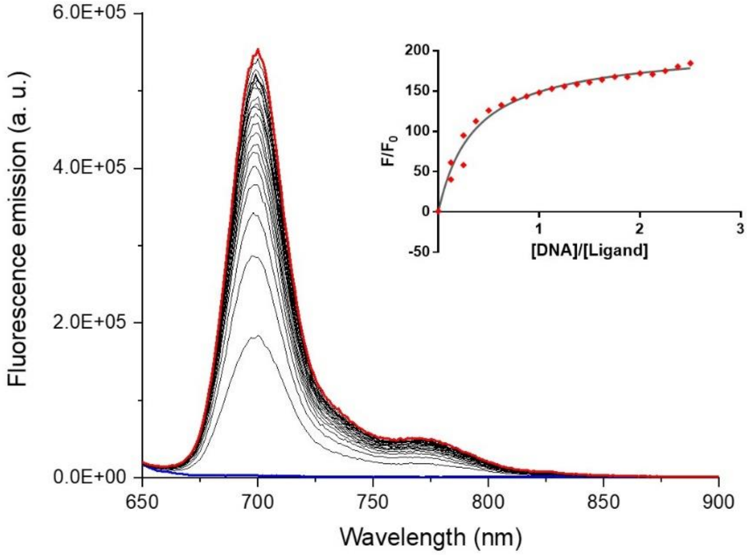

Figure S14. Fluorescence titration of **ZnPc** (2 μM) with G4 DNA CEB25 in Tris buffer (Tris 10 mM, KCl 100 mM, pH= 7.4, λ_{exc} = 620 nm). Inset: Plot of the F/Fo vs. ratio [DNA]/[MPc]. Blue spectrum corresponds to the initial titration and red spectrum to last one.

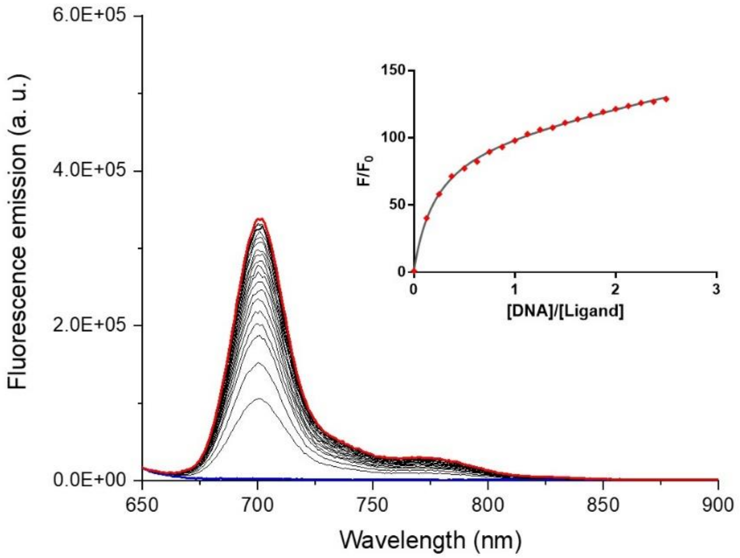

Figure S15. Fluorescence titration of **ZnPc** (2 μM) with G4 DNA Kras in Tris buffer (Tris 10 mM, KCl 100 mM, pH= 7.4, λ_{exc} = 620 nm). Inset: Plot of the F/Fo vs. ratio [DNA]/[MPc]. Blue spectrum corresponds to the initial titration and red spectrum to last one.

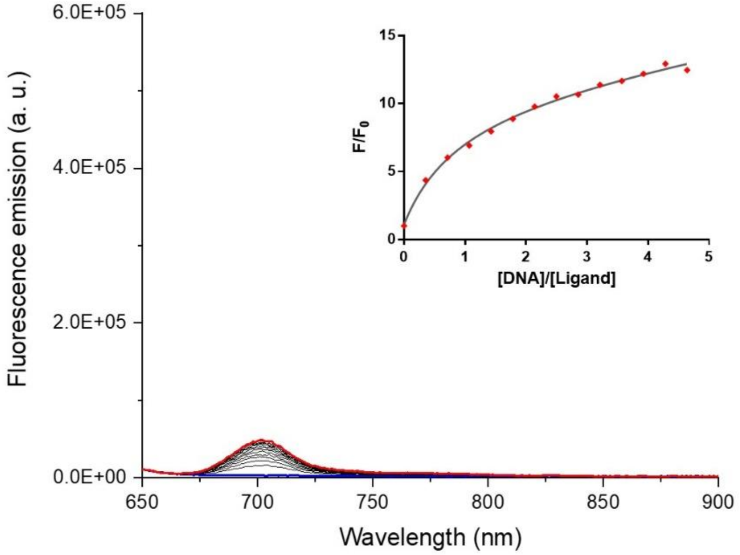

Figure S16. Fluorescence titration of **ZnPc** (2 μM) with duplex DNA ds26 in Tris buffer (Tris 10 mM, KCl 100 mM, pH= 7.4, λ_{exc} = 620 nm). Inset: Plot of the F/Fo vs. ratio [DNA]/[MPc]. Blue spectrum corresponds to the initial titration and red spectrum to last one.

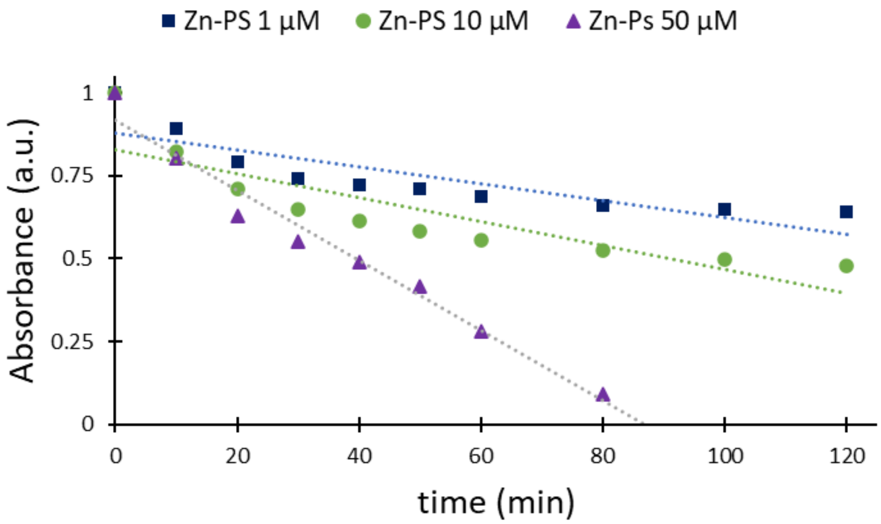

Figure S17. Decay rate of DPBF in DMSO induced by **Zn-PS** under red light irradiation.

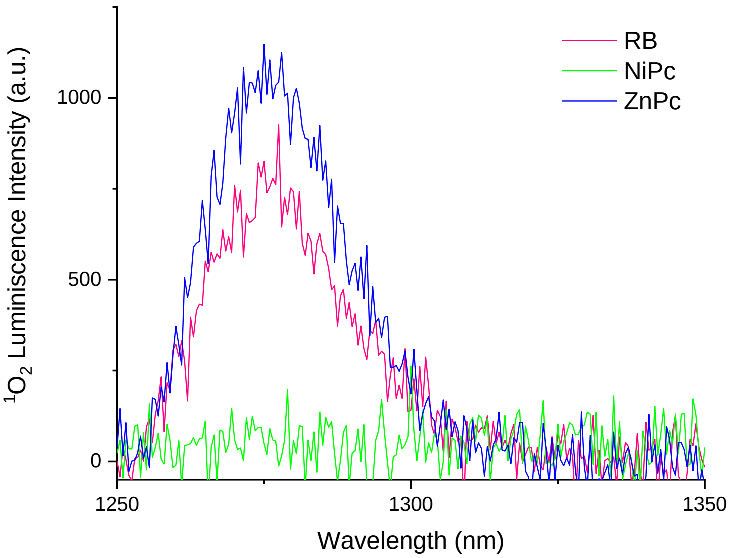

Figure S18. Luminescence of singlet oxygen generated by irradiating of **RB**, **NiPc** and **ZnPc**.

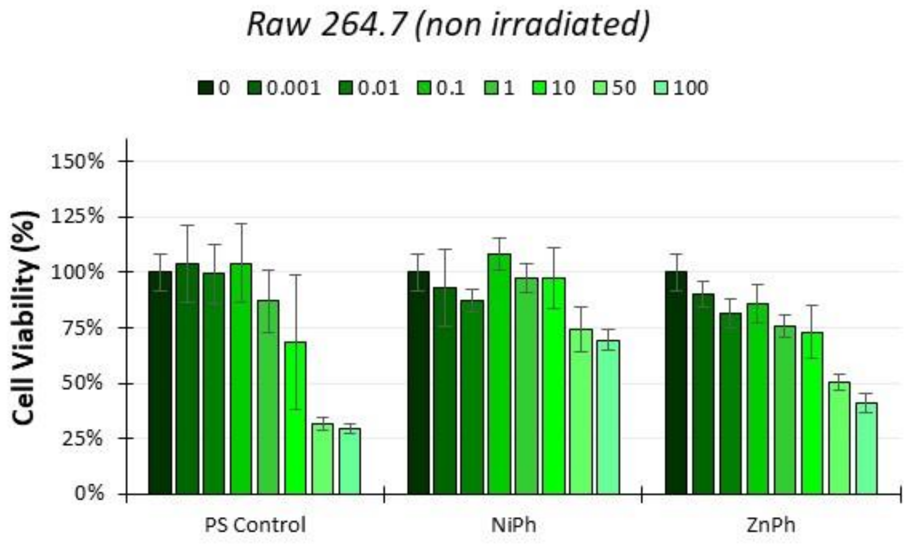

Figure S19. Cell viability on Raw264.7 cell line of **Zn-PS**, **NiPc** and **ZnPc** in the dark. Colors and numbering in the legend refer to the compound concentrations (µM).

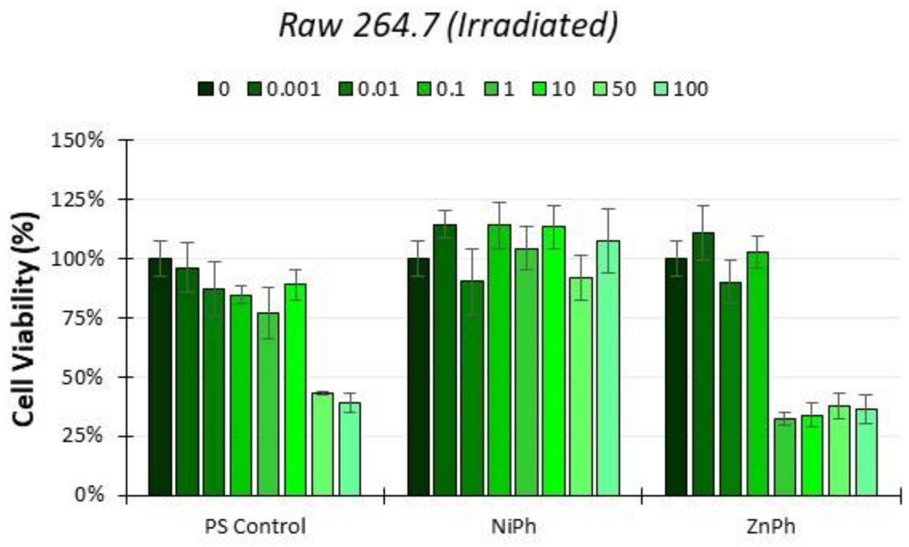

Figure S20. Cell viability on Raw264.7 cell line of **Zn-PS**, **NiPc** and **ZnPc** under irradiation. Colors and numbering in the legend refer to the compound concentrations (µM).

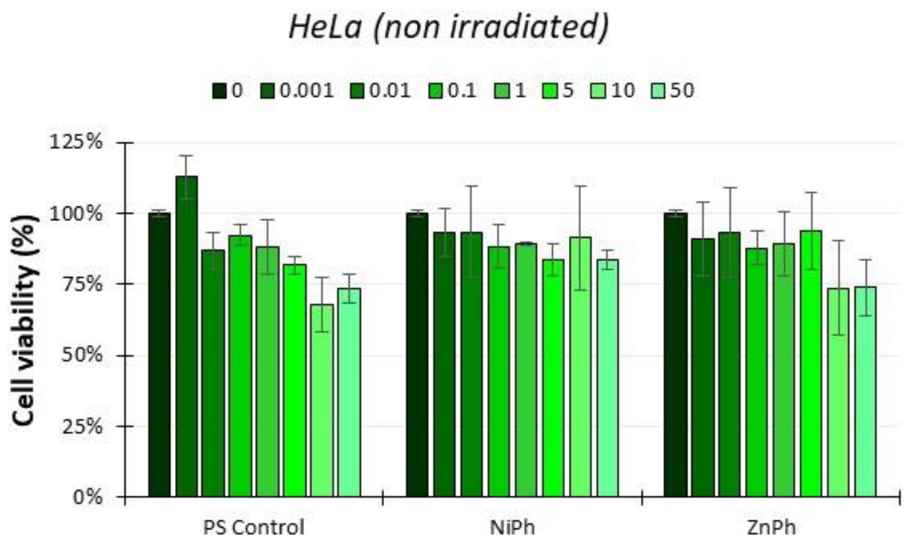

Figure S21. Cell viability on HeLa cell line of **Zn-PS**, **NiPc** and **ZnPc** in the dark. Colors and numbering in the legend refer to the compound concentrations (μM).

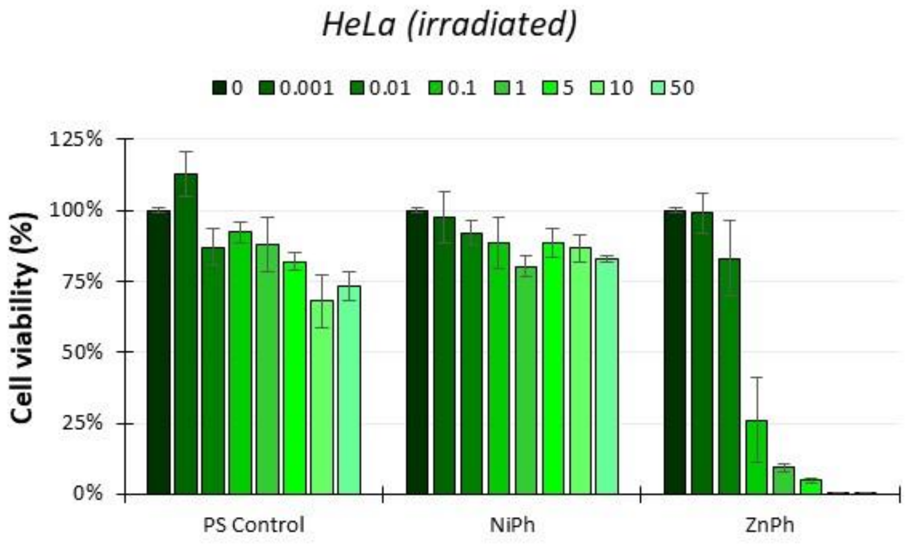

Figure S22. Cell viability on HeLa cell line of **Zn-PS**, **NiPc** and **ZnPc** under irradiation. Colors and numbering in the legend refer to the compound concentrations (μM).
